# Cytotoxicity of a Cell Culture Medium Treated with a High-Voltage Pulse Using Stainless Steel Electrodes and the Role of Iron Ions

**DOI:** 10.3390/membranes12020184

**Published:** 2022-02-04

**Authors:** Gintautas Saulis, Raminta Rodaitė-Riševičienė, Rita Saulė

**Affiliations:** Department of Biology, Faculty of Natural Sciences, Vytautas Magnus University, 58 K. Donelaičio Str., LT-44248 Kaunas, Lithuania; raminta.rodaite-riseviciene@vdu.lt (R.R.-R.); rita.saule@vdu.lt (R.S.)

**Keywords:** electroporation, electrolysis reactions, cell viability, mouse hepatoma, CHO

## Abstract

High-voltage pulses applied to a cell suspension cause not only cell membrane permeabilization, but a variety of electrolysis reactions to also occur at the electrode–solution interfaces. Here, the cytotoxicity of a culture medium treated by a single electric pulse and the role of the iron ions in this cytotoxicity were studied in vitro. The experiments were carried out on mouse hepatoma MH-22A, rat glioma C6, and Chinese hamster ovary cells. The cell culture medium treated with a high-voltage pulse was highly cytotoxic. All cells died in the medium treated by a single electric pulse with a duration of 2 ms and an amplitude of just 0.2 kV/cm. The medium treated with a shorter pulse was less cytotoxic. The cell viability was inversely proportional to the amount of electric charge that flowed through the solution. The amount of iron ions released from the stainless steel anode (>0.5 mM) was enough to reduce cell viability. However, iron ions were not the sole reason of cell death. To kill all MH-22A and CHO cells, the concentration of Fe^3+^ ions in a medium of more than 2 mM was required.

## 1. Introduction

Electroporation is an effective method for the modification of cell membrane permeability, the number of applications of which are steadily increasing in biology, oncology, genetics, immunology, and biotechnology [1,2]. There are numerous theoretical and experimental studies devoted to various aspects of this phenomenon, such as the changes of permeability of a cell plasma membrane to various substances in the presence of an electric field [3,4,5,6,7] and the restoration of the state of low permeability after treatment [7,8,9]. However, the permeabilization of cell membranes is not the only consequence of the treatment of cell suspension with pulses of a strong electric field.

When an electric current passes through a cell suspension, it causes heating (Joule heating), and various chemical reactions occur at the surface between the solution and the electrodes (electrolysis). These may include the evolution of gases, the separation of substances, the dissolution of the electrode, or the appearance of new substances in the solution [10,11,12]. The processes of electrolysis lead to changes of the temperature, pH, and the chemical composition of an experimental medium. Due to this, the efficiency of electroporation technology might depend not only on the changes created in the cell membranes, but on the electrochemical processes as well. When the processes of electrolysis are not taken into consideration, not only can the results obtained be misinterpreted [13], but it is also difficult to improve the experimental procedures.

As an example of one of the most important electrochemical processes, the dissolution of an anode (and sometimes a cathode [14]) can be mentioned. This process occurs due to the oxidation of the metal of the anode [12]. For example, the release of Al^3+^ from aluminium electrodes [13,14,15,16]; Cu^2+^ from copper electrodes [17]; and iron, chromium, and manganese ions from stainless steel electrodes [15,18,19,20] have been observed during cell electroporation experiments.

Several consequences of this process can be important for studying and/or using the cell electroporation phenomenon. Metal ions released from the electrodes can: (i) affect physiological processes [13]; (ii) change the pH of the medium [21]; (iii) reduce cell viability [15,21]; (iv) change the electric conductivity of the medium [21,22]; (v) increase the roughness of the anode surface [23]; (vi) build up complexes with various molecules, including proteins, DNA, and RNR [17,24]; as well as (vii) quench the fluorescence of fluorophores [21,25].

It must be stressed that electrolysis reactions occurring during pulses of strong electric fields accompany the permeabilization of the cell membrane. Thus, various solutes may enter or leave the cells through the permeabilized membrane [26,27,28]. Therefore, any uncontrollable changes of the physicochemical properties of a solution during the procedures of cell electromanipulation are especially undesirable. The metal ions released from the electrodes might affect some of the cellular physiological processes [13].

Currently, studying the effects of electrolysis has become especially important, as electroporation has recently been introduced for use in vivo for electrochemotherapy [29,30] and ablation with irreversible electroporation [31,32]. It has also been proposed for increasing the permeability of skin with the purpose of transdermal drug delivery [33], transformation of skin cells [34], and tissue-specific extraction of RNA and proteins [35]. The issues of electrochemical reactions are especially critical for all the applications of pulsed electric field treatment in the food industry [11,36] as well as the recently proposed Electrolytic Electroporation (the combination of electroporation with electrolysis) [37].

Earlier, it was noticed that the media treated by high-voltage electric pulses, which are usually utilized for cell electroporation or other electromanipulation (electrofusion, electroinsertion, etc.) purposes, exert some cytotoxicity [38,39,40]. Recently, a synergistic effect between electrolysis and electroporation in cell killing has been reported [41]. The cytotoxicity was attributed to the electrolytic production of free chlorine and oxygen, metal ions released from the electrodes, and possibly direct anodal oxidation of other substances [15,38,39,40,42,43]. However, the cytotoxicity of a pulse-treated medium has not been studied in enough detail yet.

It has to be kept in mind that electrodes, which are utilized in electroporation experiments, may be involved in the biochemical processes taking part in the experimental systems. Diverse electrode materials are used in commercially available and home-made electrodes, which are utilized to electroporate the cells both in vitro and in vivo. However, the most popular one is still stainless steel [15,17,18,20,29,44,45,46].

The present work is an attempt to investigate the dependence of the cytotoxicity of a cell culture medium treated by high-voltage pulses on treatment parameters and determine plausible reasons that make the cell culture medium cytotoxic. A specific aim is to elucidate the role of one of the electrochemical processes—the release of metal ions from the anode occurring during the action of high-voltage electric pulses—in this cytotoxicity. In this study, it has been shown that: (i) the cell culture medium treated with an electric pulse is much more cytotoxic when stainless steel electrodes are used in comparison with aluminium electrodes and/or a platinum anode, and (ii) only a small part of this cytotoxicity is governed by the iron ions released from the electrodes.

## 2. Materials and Methods

### 2.1. Main Materials

The cell culture medium consisted of Dulbecco‘s modified Eagle‘s medium (cat. No. D5546, Sigma-Aldrich Chemie, Steinheim, Germany), 9% fetal bovine serum—FBS (F7524, Sigma-Aldrich Chemie), 1% L–glutamine solution (G7513, Sigma-Aldrich Chemie), 100 U/mL penicillin, and 90 μg/mL streptomycin antibiotics (P0781, Sigma-Aldrich Chemie). The cell electroporation medium was Minimum Essential Medium Eagle, Spinner Modification (S-MEM) (M8167, Sigma-Aldrich Chemie). Altogether, 0.9% NaCl (Balkanpharma-Troyan AD, Troyan, Bulgaria), FeCl_2_, and FeCl_3_ (Fluka Chemie GmbH, Buchs, Switzerland) were used to prepare the solutions of 0.9% NaCl with various concentrations of Fe^2+^ and Fe^3+^.

### 2.2. Growth of Cells and Preparation of Cell Suspension

Mouse hepatoma MH–22A, rat glioma C6, and Chinese hamster ovary (CHO) cells were grown in monolayer cultures in 25 cm^2^ flasks (Greiner Bio-One, Frickenhausen, Germany) at 37 °C and 5% CO_2_ in the incubator IR AutoFlow NU-2500E (NuAire, Plymouth, MN, USA). When estimating the fraction of electroporated cells, the cells were grown in 75 cm^2^ (250 mL) flasks (Greiner Bio-One). All manipulations that required sterile conditions were performed in a vertical laminar flow cabinet Aura Vertical SD4 (BIOAIR Instruments, Siziano, Italy).

When cells reached confluence, they were detached from the flask bottom by using 2 mL of a 0.25% trypsin–0.02% ethylenediaminetetraacetic acid (EDTA) solution (T4049, Sigma-Aldrich Chemie). The cells were trypsinized under constant verification, and as soon as they had detached from the flask bottom, typically after 2–5 min, trypsin was inactivated by adding 2 mL of the culture medium. After centrifugation of the suspension for 5 min at 1000 rpm (~380× *g*, rotor R-12/15, LMC-300, Biosan, Riga, Latvia), cells were resuspended in an S-MEM at a concentration of approximately 1 × 10^6^ cells/mL.

When estimating the fraction of electroporated cells, cells were resuspended in the culture medium at approximately 2–5 × 10^7^ cells/mL and kept for 60–70 min at room temperature (20–21 °C). During this time, the cells restored the normal level of the intracellular concentration of potassium ions [47]. Then, the cells were electroporated within 15–20 min.

### 2.3. Electric Pulse Generator and Electrodes Used to Treat Medium or Cell Suspension

To treat cell culture medium or cell suspension with high-voltage pulses, a BTX ECM 2001 Electro Cell Manipulator (Harvard Apparatus, Holliston, MA, USA) was used. The shape and parameters of the pulse were monitored with the oscilloscope TBS 1072B-EDU (Tektronix U.K. Ltd., Berkshire, UK) or analogue storage oscilloscope S8-13 (Vilnius Factory of Radio Measuring Devices, Vilnius, Lithuania) and a 1/10 voltage divider [48].

The electrodes were made from stainless steel plates. Plate dimensions were 50 mm (length), 10 mm (width) and 1 mm (thickness). The gap between the electrodes was 2 mm. The main constituents of stainless steel used to manufacture the electrodes were (average ±2 × standard deviation (SD), %): iron (69.87 ± 0.14), chromium (17.582 ± 0.075), nickel (10.28 ± 0.10), manganese (0.801 ± 0.055), titanium (0.80 ± 0.02), cobalt (0.294 ± 0.075), copper (0.19 ± 0.03), and molybdenum (0.070 ± 0.003). This corresponds to the austenitic family of stainless steel of the 300 series.

When evaluating the influence of the electrode material on the medium cytotoxicity, to avoid the release of iron, chromium, nickel, and manganese ions from the stainless steel anode, the anode was completely covered with a platinum foil. In addition, commercially available cuvettes with aluminum alloy electrodes CUV-02 (Cyto Pulse Sciences, Inc., Columbia, MD, USA) with the inter-electrode distance of 2 mm were used. The aluminum alloy contained small amounts of other metals (average ± 2SD, %): iron (0.139 ± 0.017), chromium (0.110 ± 0.006), titanium (0.048 ± 0.006), vanadium (0.037 ± 0.009), and copper (0.026 ± 0.004).

The composition of stainless steel and aluminum alloy was determined using an X-ray fluorescence analysis (XRF) method with the spectrometer Niton^TM^ XL3t 980 GOLDD+ XRF Analyzer (Thermo Fisher Scientific, Waltham, MA, USA) equipped with a silicon drift detector.

### 2.4. Treatment of the Culture Medium with an Electric Pulse

A 50 µL droplet of the cell culture medium was placed between a pair of flat electrodes, and a single square-wave electric pulse with a duration of 100, 500, or 2000 µs and an amplitude ranging from 20 to 500 V was applied. These voltages corresponded to the electric field strengths ranging from 0.1 to 2.5 kV/cm.

The pulse-treated sample of the cell culture medium was collected into 2 mL Eppendorf tubes, and the procedure was repeated until the amount of pulse-treated medium required for seeding the cells was collected (2 mL for each Petri dish). To avoid any influence on the cell viability of the increased medium temperature, which might occur due to the application of the pulse [49,50], after the exposure, the medium was allowed to reach room temperature before seeding the cells.

The values of the total charge that has flown through the sample, *C*, were calculated from the equation:

*C* = *U t*_imp_*/R* (1)

where *U* is the voltage of an electric pulse, *t*_imp_—pulse duration, and *R*—sample resistance (~50 Ω).

### 2.5. Treatment of the Cell Suspension with an Electric Pulse

A 50 µL droplet of the cell suspension was placed between a pair of flat electrodes and subjected to a single square-wave electric pulse. Pulses with durations of 100, 500, or 2000 µs and an amplitude ranging from 40 to 500 V (the electric field strengths from 0.2 to 2.4 kV/cm) were used.

### 2.6. Determination of the Fraction of Electroporated Cells

Cells usually contain high concentrations of potassium ions (K^+^). Pore formation in the plasma membrane results in their release. Thus, the fraction of electroporated cells can be determined from the extent of the release of intracellular K^+^ ions [47].

After an electric pulse was applied to the cell suspension, cells were immediately transferred to a chilled Eppendorf tube, kept on ice for 5–10 min, and then kept for 30–40 min at 10–11 °C to prevent pores from closing and to allow equilibration between intracellular and extracellular K^+^ concentrations. The extracellular potassium concentration was measured by means of a mini K^+^-selective and reference electrodes [47].

### 2.7. Determination of the Fraction of Cells Killed by the Electric Pulse

After the exposure to an electric pulse, 40 μL of the cell suspension was diluted with 1.56 mL of a warm (37 °C) Minimum Essential Medium Eagle, Spinner Modification, and incubated at 37 °C to allow pores to reseal. After 10 min, 100 µL of the cell suspension was diluted with 0.5 mL of S-MEM (37 °C), and 100 μL of the final cell suspension was seeded in duplicate or triplicate (200–300 cells per Petri dish) into 34 mm internal diameter (9.2 cm^2^ growth surface) Petri dishes for the subsequent determination of their viability. Petri dishes (93040, TPP Techno Plastic Products AG, Trasadingen, Switzerland) contained 2 mL of the cell growth medium (culture medium, which was additionally supplemented with 90 U/mL penicillin and 90 μg/mL streptomycin). The total dilution of the cell suspension treated by the electric pulse was 5040-fold.

### 2.8. Influence of an Electric Pulse-Treated Medium, its pH, and Fe^2+^ or Fe^3+^ Ions on Cell Viability

For studying the influence of the Fe^2+^ or Fe^3+^ ions and the culture medium pre-treated with an electric pulse on the cell viability, 40 mL of the cell suspension was diluted with 1.46 mL of a Minimum Essential Medium Eagle, Spinner Modification (37 °C). After the additional dilution of 100 mL of the cell suspension with 0.7 mL of S-MEM (37 °C), finally, the cells were seeded into 34 mm internal diameter (9.2 cm^2^ growth surface) Petri dishes (93040, TPP Techno Plastic Products AG) containing 2 mL of the culture medium.

The medium in these Petri dishes was: (i) treated with an electric pulse, (ii) its pH was adjusted by adding an appropriate amount of HCl, or (iii) supplemented with various concentrations of FeCl_2_ or FeCl_3_. The number of cells seeded in each dish was about 200–300 cells. The Petri dishes with cells were incubated at 37 °C and 5% CO_2_ in the incubator for 5–6 (rat glioma C6 and Chinese hamster ovary cells) or 9–10 (mouse hepatoma MH-22A cells) days for the determination of cell viability.

### 2.9. Determination of Cell Viability

Cell viability was determined by means of a colony-forming assay [51]. Although colony formation assay is time-consuming and labor intensive, particularly when many samples are being processed, it is the most consistent, relevant, and reproducible viability assay in vitro [52].

After seeding the cells into Petri dishes, they were incubated at 37 °C and 5% CO_2_ in a water-jacketed incubator IR AutoFlow NU-2500E (NuAire, Plymouth, MN, USA). Petri dishes were made of polystyrene. To enhance the adhesion of the cells to the plastic surface, the manufacturer (TPP) activated the growth areas of dishes by an optic-mechanic method developed by them. After incubation for 6–7 (C6 and CHO cells) or 9–10 (MH-22A cells) days, the formed colonies were fixed with 96% ethanol (Stumbras, Kaunas, Lithuania), stained with a Gram’s crystal violet solution (Fluka Chemie, Buchs, Switzerland), and counted under stereo microscope MBS-9 (LOMO, St. Petersburg, Russia) or OZL 463 (Kern & Sohn GmbH, Balingen, Germany). The viability of cells was calculated as the percentage of the colonies obtained from the untreated control cells:

*Viability (%)* = *N*_sample_/*N*_control_, (2)

where *N*_sample_ and *N*_control_ are the average numbers of the colonies in Petri dishes in which treated and untreated control cells were seeded, respectively.

Only the colonies containing more than 50 individual cells were counted [52]. The average colony count for the two to three dishes was used to calculate the fraction of viable cells.

### 2.10. Determination of the Amount of Iron Ions (Fe^2+^ & Fe^3+^) Released into the Medium

A 50 mL droplet of a 154 mM NaCl solution was placed between two stainless steel electrodes (inter-electrode distance was 2 mm) and subjected to a single square-wave electric pulse. The duration of the pulse was 2 ms, and the amplitude was varied from 0.2 to 1.2 kV/cm. The procedure was repeated several times to collect at least 1 mL of the pre-treated medium. Then, the total concentration of iron ions (Fe^2+^ & Fe^3+^) in the medium was determined by the thiocyanate method [21]. The optical density of the medium was measured at 474 nm by using the spectrophotometer Genesys 10 (Thermo Fisher Scientific, Waltham, MA, USA).

### 2.11. Statistical Analysis

Experimental points are averages from 3–4 experiments ± SD. To fit the experimental dependences obtained here, the software SigmaPlot 11.0 (Systat Software, Point Richmond, CA, USA) was used.

The dependences of the fraction of electroporated cells, *F*(*E*_0_), on the pulse amplitude, *E*_0_, were fitted with a three-parameter sigmoid curve [53]:

(3) $$F(E_{0})=\frac{F_{\max}}{1+\exp[(E_{C}-E_{0})/b]},$$

where *F*_max_ is the maximal value of the fraction of electroporated cells, *E*_C_ is the amplitude of the electric pulse at which *F*(*E*_C_) = *F*_max_/2, and *b* controls how steeply the curve rises. Parameters *F*_max_, *E_C_*, and *b* were determined by minimizing a residual sum of squares.

The dependences of the cell viability on the concentration of iron ions (Fe^2+^ or Fe^3+^) were fitted with a three-parameter logistic curve:

(4) $$Viability=\frac{Viability_{\max}}{1+{\left([Fe^{2+/3+}]/EC_{50}\right)}^{-Hillslope}},$$

where *Viability*_max_ is the maximum value of the cell viability (the minimum value was chosen equal to 0), [Fe^2+/3+^] is the concentration of iron ions, *EC*_50_ is the point on the curve halfway between 0 and *Viability*_max_, and *Hillslope* is Hill’s slope of the curve, which defines the steepness of the curve at [Fe^2+/3+^] = *EC*_50_. The unknown parameters *Viability*_max_, *EC_50_*, and *Hillslope* were determined by minimizing a residual sum of squares.

## 3. Results and Discussion

Exposure of cells by a short (nanoseconds–milliseconds) pulse of a strong electric field causes not only an increase of the membrane permeability to ions [54,55,56] or larger molecules [26,57,58], but also death of some part, sometimes a substantial one, of treated cells [53,59,60,61,62]. The death of the cells treated by pulsed electric fields is a well-known phenomenon, and it is considered that the main reason of cell death is the permeabilization of the cell plasma membrane [63,64,65]. However, there are data showing that other factors, e.g., the products of electrochemical reactions, can also contribute to cell death [15,38,39,40,42], and all plausible factors have not been established yet [66].

Several studies reported that media treated with high-voltage electric pulses, which are usually utilized for cell electroporation or other electromanipulation (electrofusion, electroinsertion, etc.) purposes, demonstrate cytotoxic and bactericidal features [38,39,40]. The toxicity of a pulse-treated medium was attributed to the electrolytic production of free chlorine and oxygen, metal ions released from the electrodes, and possibly direct anodal oxidation of other substances [15,38,39,40,42]. However, the detailed dependences on the treatment parameters, such as the strength of an electric field and its duration as well as all the reasons causing the cytotoxicity of the electric pulse-treated medium, still need to be determined.

### 3.1. Influence of an Electric Pulse on the Integrity of the Cell Plasma Membrane (Electroporation) and Cell Viability

To study the role of a pulse-treated medium as one of the plausible factors causing cell death after the exposure of the cell suspension to a strong electric field, first, the ability of the pulse of a strong electric field to electroporate and kill the cell has been studied. For this, the influence of a single square-wave electric pulse on the integrity of the cell plasma membrane (electroporation) and the cell viability was investigated on pulse parameters, such as the strength of the applied electric field and its duration. The experiments have been carried out on mouse hepatoma MH-22A cells. The cell suspension was treated with a single square-wave electric pulse, and the fractions of electroporated and viable cells were determined.

Here, the cell was considered as electroporated when its plasma membrane became permeable to small potassium ions (K^+^) [67], and the fraction of electroporated cells was estimated from the extent of the release of the intracellular potassium ions [47]. This method allows for the detection of just a few small pores in the cell plasma membrane.

The cell viability was evaluated by a colony-forming assay [53]. In the latter case, to minimize the plausible influence of the medium treated by an electric pulse on the cell viability, after the pulse, the cell suspension was, as quickly as possible (usually within 15–20 s), diluted substantially (~40-fold) with the electroporation medium (S-MEM). Then, after a 10-min incubation at 37 °C, the suspension was diluted once again 6-fold with S-MEM before finally seeding the cells into Petri dishes with the additional 21-fold dilution with the cell culture medium (see Section 2 Materials and Methods). This way, the total dilution of the cell suspension treated by the electric pulse was 5040-fold. Therefore, it can be considered that when estimating the electric pulse killing ability, the pulse-treated medium itself was not making a noticeable influence on the cell viability.

**Figure 1:**
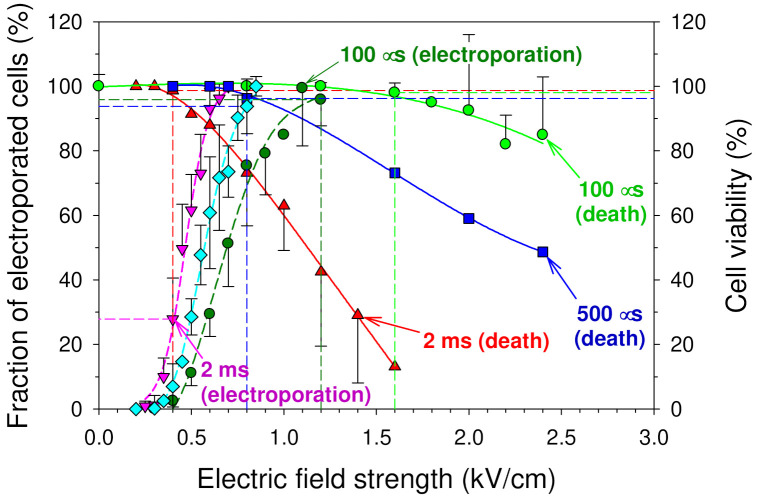
The dependence of the fractions of electroporated (left ordinate) and viable (right ordinate) mouse hepatoma MH-22A cells on the electric field strength of a single square-wave pulse. These dependences are shown for pulses with a duration of 100 μs, 500 μs, and 2 ms. The fraction of electroporated cells was estimated from the increase of the permeability of the cell plasma membrane to intracellular potassium ions [47]. The viability of the cells treated with electric pulses was evaluated by a colony-forming efficiency assay, and it was calculated as the percentage of the colonies obtained from the untreated control cells. The data for pulses with the duration of 100 μs and 2 ms were taken from Saulis and Saule [53]. Experimental points are averages from 3–4 experiments ± SD.

In Figure 1, the fractions of pulse-treated cells, which were exposed to a single square-wave electric pulse but remained viable, are shown as the functions of the pulse amplitude (electric field strength). The pulses with the duration of 100, 500, and 2000 μs were used. It can be seen from this figure that increasing either the electric field strength (at constant pulse duration) or the duration of the pulse (at constant electric field strength) decreased the cell viability (increased the fraction of the cells, which were killed as a consequence of the exposure to an electric pulse). It is considered that one of the main reasons of cell death after PEF treatment is the increased permeability or even substantial structural damage of the cell plasma membrane, causing the release of intracellular substances out of the cell [68] and/or osmotic swelling [69,70,71].

Usually, some part, sometimes a substantial one, of electroporated cells survives. As a result, there is often some difference between the fractions of electroporated and the cells killed by the electric treatment [53,72]. For the comparison, the dependences of the fraction of mouse hepatoma MH-22A cells, which were electroporated by a single square-wave electric pulse of different durations (100 and 500 µs as well as 2 ms), on the pulse amplitude are also shown in Figure 1. The cell membrane electroporation was determined by measuring the changes in the permeability of the cell plasma membrane to small potassium ions (K^+^) [47]. Each experimental point is the mean of three to four independent measurements ± SD. The data for pulses with a duration of 100 µs and 2 ms were taken from Saulis and Saule [53].

In Figure 1, it is clearly seen that the “cell death” curves are significantly shifted toward the higher pulse amplitudes in comparison to the “electroporation” ones. This means that to create small pores in the cell plasma membrane is not enough to kill the cells. Stronger pulses are required to kill the cells. For example, the electric pulse with the duration of 2 ms and the amplitude of 0.4 kV/cm electroporated almost 30% of cells, but only about 1% of cells were killed by such a pulse (see red dashed vertical line in Figure 1). The electric pulse with the same duration and an amplitude of 0.7 kV/cm electroporated 100% of the cells, but only about 20% of them were killed by such a pulse. The majority (80%) of electroporated cells had survived after the exposure by the electric pulse with a duration of 2 ms and an amplitude of 0.7 kV/cm (Figure 1).

In the case of shorter pulses, this difference was even larger. For example, the first dead cells (~4%) appeared only when the amplitude of a 500 µs-duration pulse reached 0.8 kV/cm. Meanwhile, such a pulse electroporated 94% of cells. Pulses of a 100 µs duration with an amplitude of 1.1–1.2 kV/cm electroporated 96–100% of cells; however, all these cells survived such a treatment (Figure 1).

One of the main reasons of a better killing ability of longer pulses is most likely due to the differences in the size of the pores created by long and short pulses. Theoretical analysis [6,60] and experimental data [53,73] show that in the case of longer pulses, not only are larger pores created, but also these pores persist longer [53].

### 3.2. Influence of the Cell Culture Medium Treated by an Electric Pulse on the Cell Viability

When a high-voltage pulse is applied to the electrolyte solution with cells, besides a cell membrane permeabilization, a variety of electrolysis reactions occur at the electrode–solution interfaces [10,12]. To elucidate the influence of the products of these electrochemical reactions on the cell viability, mouse hepatoma MH-22A cells were seeded into the Petri dishes containing 2 mL of the culture medium pre-treated with a single square-wave electric pulse. The medium temperature can increase due to the application of the pulse [49,50]. To avoid this, after the exposure to an electric pulse, the medium was allowed to reach room temperature before seeding the cells into Petri dishes.

**Figure 2:**
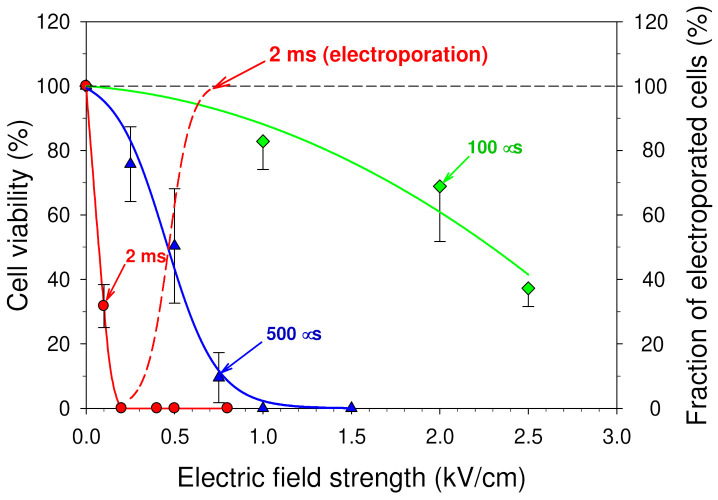
The cytotoxicity of a culture medium pre-treated with a single square-wave electric pulse. The viability of mouse hepatoma MH-22A cells (ordinate) is presented as a function of electric field strength (abscissa). The cells were grown for 9–10 days in the medium pre-treated with a square-wave electric pulse with a duration of 100 μs, 500 μs, or 2 ms. The electrodes (both the anode and the cathode) made of stainless steel were used in all experiments. The cell viability was estimated by a colony-forming assay. The dashed line shows the dependence of the fraction of cells, which were electroporated by a single 2 ms duration pulse (data are taken from Figure 1).

The obtained dependences of the mouse hepatoma MH-22A cell viability on the amplitude of a single electric pulse with a duration of 100 and 500 µs as well as 2 ms are shown in Figure 2. Each experimental point in this figure is a mean of three independent measurements ± SD.

The results obtained show strong cytotoxic effect of the cell culture medium treated by a single electric pulse with a duration of 100 µs–2 ms and an amplitude of 0.2–2.5 kV/cm. These pulse parameters are within the range of the pulses, which are usually utilized for cell electroporation in vitro [27,39] as well as gene delivery [74] and electrochemotherapy [75] in vivo.

Half of the cells (50%) survived in the medium pre-treated with a pulse at an amplitude of 0.5 kV/cm and a duration of 500 ms (Figure 2). However, when the amplitude of an electric pulse was increased to 1 kV/cm, none of the cells had survived. Medium treated with a longer electric pulse (2 ms) was much more cytotoxic. Only 32% of mouse hepatoma MH-22A cells survived in the medium pre-treated with a pulse with an amplitude of 0.1 kV/cm and a duration of 2 ms (Figure 2). None of the cells survived in the medium treated with a slightly stronger pulse at 0.2 kV/cm.

Usually, it is considered that the main reasons of cell death after PEF treatment are the increased permeability or even substantial structural damage of the cell membrane [68,69,70,71]. However, in our experiments, cells were not exposed to an electric pulse at all. In addition, it could be seen from Figure 1 that even in the case when the cells were exposed to an electric pulse with an amplitude of 0.2 kV/cm and a duration of 2 ms, the plasma membranes of all cells remained intact. The dashed line in Figure 2 is taken from Figure 1, and it shows the dependence of the fraction of cells, which were electroporated (permeable to K^+^) by a single 2 ms duration pulse.

### 3.3. Dependence of the Killing Ability of an Electric Field Pulse and the Medium Treated with the Pulse on the Electric Charge That has Flown through the Sample

The next aim was to ascertain that the consequences of electrolysis reactions were responsible for the cytotoxicity of the medium treated with an electric pulse. For this, the dependences of the fraction of the cells, which died in the medium treated with a square-wave electric pulse, *F*_dead_, on the charge, which has flown through the chamber with the cell suspension, were examined. Such data were extracted from the data shown in Figure 2. The values of the total charge that has flown through the sample, *C*, were calculated from Equation (1). As the cytotoxicity of substances usually depends on their concentrations, instead of the total charge, its value per unit volume of the medium, *C*_V_, were used in the graph.

**Figure 3:**
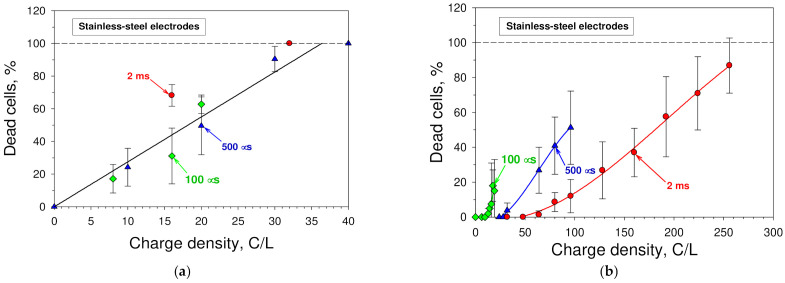
Killing of cells by the electric pulse-treated cell culture medium and the pulse of an electric field. (**a**) Dependence of the fraction of intact MH-22A cells, which died in the medium treated with a square-wave electric pulse, on the charge that has flown through the cell suspension per unit volume of the medium, obtained from the data shown in Figure 2. (**b**) Dependence of the fraction of MH-22A cells killed by a square-wave electric pulse on the charge that has flown through the cell suspension per unit volume of the medium, obtained from the data presented in Figure 1. In both cases, experiments were carried out with mouse hepatoma MH-22A cells, and their viability was determined by a colony-forming assay after the incubation at 37 °C for 9–10 days.

The dependences obtained for pulses of different duration are plotted in Figure 3a. It can be seen from these dependences that, irrespective of the pulse duration (0.1, 0.5, or 2 ms), the experimental points obtained fall around the straight line (the correlation coefficient is 0.942). The values of the treatment intensity at which all cells are killed were in the range of 30–40 C/L.

For the comparison, the dependences of the fraction of cells killed by a square-wave electric pulse on the charge that has flown through the cell suspension per unit volume of the medium were plotted for pulses of different durations (0.1, 0.5, or 2 ms) (Figure 3b). These dependences were obtained from the data presented in Figure 1 (medium volume was 50 mL). They clearly demonstrate that the main factor responsible for the death caused by the exposure of the cell suspension with the pulse of a strong electric field is not the total charge, which has flown through the cell suspension.

The dependences plotted in Figure 3a give us the opportunity to predict the cytotoxicity of the cell culture medium treated with a square-wave electric pulse toward mouse hepatoma MH-22A cells in the cases when stainless steel electrodes are used. The medium cytotoxicity can be estimated from the following equation:

(5) $$F_{dead}=aC/V_{m}$$

where *F_dead_* is the fraction of dead cells (in percent), *a*—the slope (angular coefficient) of the straight line (*a* = 2.7495), *C*—the total charge that has flown through the sample (Equation (1)), and *V_m_*—the medium volume (50 mL in our case).

### 3.4. Plausible Reasons of the Cytotoxicity of the Cell Culture Medium Treated with the Electric Pulse

To determine plausible reasons that make the cell culture medium cytotoxic when exposed to an electric pulse, basic processes occurring in the medium during electric treatment and after it should be taken into consideration. Various oxidation and reduction half-reactions can occur at the electrode—solution interfaces when an electric current passes through a water solution [11].

Two cathodic half-reactions can occur: (i) reduction of the cations, which are present in the solution, e.g., K^+^, Na^+^, etc., or (ii) reduction of water molecules (or hydrogen ions in acidic solutions) [12]. The species with the most favorable reduction potential is reduced. Solutions, which are generally utilized in cell electromanipulation procedures, contain mainly the salts of sodium, potassium, calcium, and magnesium. As the ions of Na^+^, K^+^, Ca^2+^, and Mg^2+^ are more difficult to reduce than water [6], the water reduction reaction usually takes place at the cathode:

(6) $$2H_{2}O+2e^{-}\Rightarrow H_{2}(gas)+2OH^{-}(aq)\;\;\;(E_{\mathrm{red}}=-0.8277\;V)$$

Here, the abbreviations “gas” and “aq” mean gaseous and aqueous, respectively. In parentheses, the standard reduction electrode potential of the half-reaction *E*^o^_red_ is given in reference to the standard hydrogen electrode (SHE).

There are three possible anodic half-reactions that can occur when an electric current passes through an electrolyte solution. First, the oxidation of water molecules [11,12]:

(7) $$2H_{2}O\to O_{2}\;(gas)\;+\;4H^{+}\;(aq)\;+4e^{-},\;\;\;{E^{o}}_{\mathrm{ox}}=-1.229\;V\;(E_{\mathrm{red}}=+1.229\;V),$$

Second, the oxidation of the anion of the solute, e.g., Cl^-^ or OH^-^ [11,12]:

(8) $$2Cl^{-}(aq)\to Cl_{2}\;(gas)\;+\;2e^{-},\;\;\;{E^{o}}_{\mathrm{ox}}=-1.358\;V\;({E^{o}}_{\mathrm{red}}=+1.358\;V),$$

(9) $$4OH^{-}(aq)\to O_{2}\;(gas)\;+\;2H_{2}O+4e^{-},\;\;\;{E^{o}}_{\mathrm{ox}}=-0.401\;V\;({E^{o}}_{\mathrm{red}}=+0.401\;V).$$

Third, the oxidation of the metal of the electrode [11,12]:

(10) $$Fe\;(s)\to Fe^{2+}\;(aq)\;+\;2e^{-},\;\;\;{E^{o}}_{\mathrm{ox}}=+0.441\;V\;({E^{o}}_{\mathrm{red}}=-0.441\;V)$$

(11) $$Fe\;(s)\to Fe^{3+}\;(aq)\;+\;3e^{-}\;\;\;{E^{o}}_{\mathrm{ox}}=+0.036\;V\;({E^{o}}_{\mathrm{red}}=-0.036\;V)$$

(12) $$Cr(s)\to Cr^{2+}(aq)+2e^{-}\;\;\;{E^{o}}_{\mathrm{ox}}=+0.90\;V\;({E^{o}}_{\mathrm{red}}=-0.90\;V)$$

(13) $$Ni(s)\to Ni^{2+}(aq)+2e^{-}\;\;\;{E^{o}}_{\mathrm{ox}}=+0.257\;V\;({E^{o}}_{\mathrm{red}}=-0.257\;V)$$

(14) $$Mn(s)\to Mn^{2+}(aq)+2e^{-}\;\;\;{E^{o}}_{\mathrm{ox}}=+1.185\;V\;({E^{o}}_{\mathrm{red}}=-1.185\;V)$$

(15) $$Al(s)\to Al^{3+}(aq)+3e^{-}\;\;\;{E^{o}}_{\mathrm{ox}}=+1.66\;V\;({E^{o}}_{\mathrm{red}}=-1.66\;V)$$

Here, the abbreviation “s” means solid and anodic half-reactions are written as oxidation reactions, and the standard oxidation electrode potentials *E*_ox_ are given relative to the standard hydrogen electrode (SHE). As the sign of the potential of the reaction depends on whether an electrochemical reaction is written as an oxidation or a reduction, here in parentheses, the standard reduction electrode potentials of half-reactions *E*_red_ are also given in reference to SHE.

When a consumable (non-inert) electrode is used, e.g., the one made from stainless steel or aluminum, usually, the most favorable anodic half-reaction is the oxidation of the atoms of the electrode [11]. Besides iron, stainless steel contains chromium, copper, nickel, manganese, and other metals. Thus, as a result of reactions (10)–(14), ferric and/or ferrous, chromium, nickel, and manganese ions are released from the anode into the solution. Dissolution of stainless steel electrodes has been reported in a number of cell electroporation [13,14,15,17] or food processing [18,45,46,76] experiments.

It can be assumed that metal ions (mainly iron, chromium, and nickel [45]) released from the stainless-steel anode can be, at least in part, responsible for the cytotoxicity of the pulse-treated medium [15]. For example, free or ineffectively sequestered iron, especially when it is present in excess, can be very toxic to cells. Under aerobic conditions, it catalyzes the reactions, which lead to the production of toxic oxygen radicals [77]. Several groups studying the cell electroporation phenomenon have demonstrated the cytotoxicity of iron ions [15,21].

### 3.5. Influence of the Electrode Material on the Cytotoxicity of the Culture Medium Treated with an Electric Pulse

**Figure 4:**
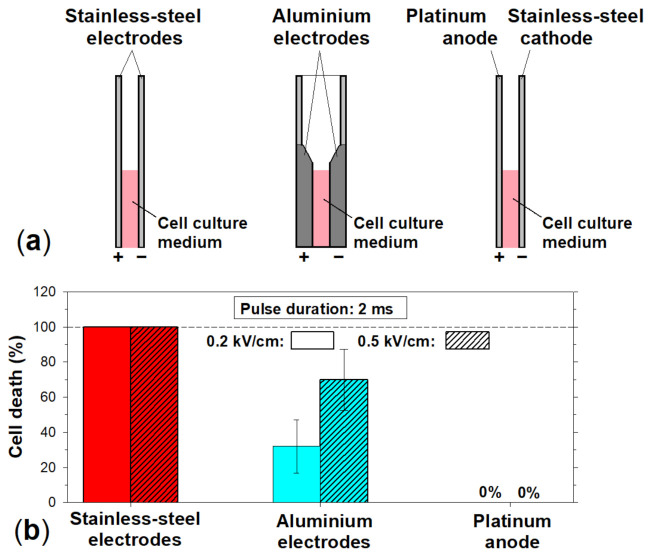
Influence of the electrode material on the cytotoxicity of the culture medium treated with an electric pulse. (**a**) Three different combinations of electrodes that were used: (1) stainless steel anode and cathode, (2) aluminum anode and cathode, and (3) platinum anode and stainless steel cathode. (**b**) The fraction of the mouse hepatoma MH-22A cells, which died in the medium treated with a square-wave electric pulse. In all cases, the pulse duration was 2 ms and the amplitude was 0.2 and 0.5 kV/cm. The cell viability was estimated by a colony-forming assay.

The next step was to determine what particular primary electrochemical reactions and/or secondary and tertiary chemical ones are responsible for the cytotoxicity of a pulse-treated medium. This could be accomplished by studying the influence of electrode material on the medium cytotoxicity. Here, three different combinations of electrodes were compared (Figure 4a): (i) stainless steel anode and cathode (used throughout all the previous experiments described here), (ii) aluminum anode and cathode, and (iii) platinum anode and stainless steel cathode.

**Figure 5:**
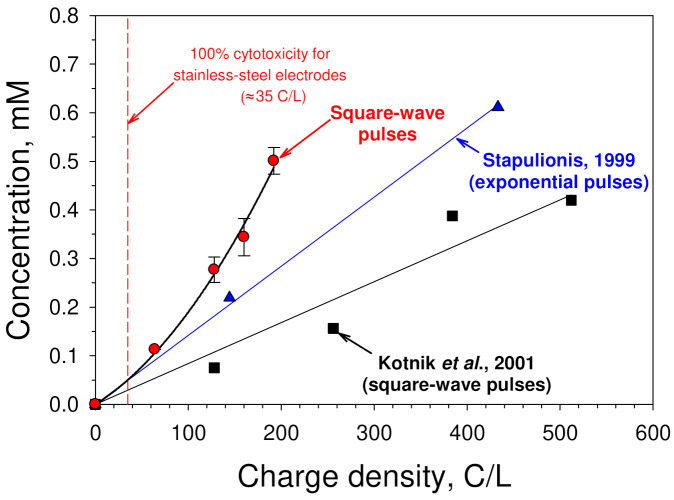
Release of iron ions from stainless steel electrodes. The total concentration of iron ions (Fe^3^^+^ and Fe^2^^+^) released from the stainless steel anode as a function of the charge that has flown through the sample per unit volume of the medium. Single square-wave electric pulses with a duration of 2 ms were used (red circles). The highest amplitude of the pulses was 240 V, which corresponded to the electric field strength of 1.2 kV/cm. The solution was 154 mM NaCl. For comparison, some of the results reported by other researchers [15,17] are shown here as well.

The fraction of the mouse hepatoma MH-22A cells, which died in the cell culture medium treated with a square-wave electric pulse, was determined. In all cases, the pulse duration was 2 ms and the amplitude was 0.2 and 0.5 kV/cm (electric charge densities of 32 and 80 C/L, respectively). The cell viability was estimated by a colony-forming assay. Recall that in the case of stainless steel electrodes, all cells died in the medium treated with such pulses (see Figure 2 and Figure 5b).

Then, the cytotoxicity of the medium treated with an electric pulse was evaluated when both stainless steel electrodes (anode and cathode) were replaced by the ones made of aluminum alloy. Commercially available cuvettes CUV-02 (Cyto Pulse Sciences, Inc., Columbia, MD, USA) with the same inter-electrode distance (2 mm) were used. Although aluminum alloy does contain other metals, such as, iron, chromium, titanium, vanadium, and copper (see Section 2 Materials and Methods), their amounts are low (e.g., 0.139 ± 0.017 and 0.110 ± 0.006 percent (±2s) for iron and chromium, respectively).

The results obtained are shown in Figure 4b. It can be seen that the pulse-treated cell culture medium was still cytotoxic to mouse hepatoma MH-22A cells when aluminum electrodes were used. However, significantly less MH-22A cells died in the pulse-treated medium, especially in the case of the pulse with the amplitude of 0.2 kV/cm (electric charge density of 32 C/L) (Figure 4b).

When using aluminum electrodes, 70% of cells died in the cell culture medium treated with a 2 ms duration and 0.5 kV/cm amplitude pulse (electric charge density is equal to 80 C/L) (Figure 4b). To achieve the same level of cytotoxicity using stainless steel electrodes, a 500 ms duration and 0.58 kV/cm amplitude pulse would be enough (electric charge density of 27.2 C/L) (Figure 2). Thus, to achieve the same level of the medium cytotoxicity, about three times stronger pulses had to be used when using aluminum electrodes in comparison with stainless steel ones. It should be recalled here that in the case of aluminum electrodes, aluminum ions are released not only from the anode but from the cathode as well [14].

Thus, to determine which processes—anodic or cathodic—were responsible for the cytotoxicity when stainless steel electrodes were used, the stainless steel anode was replaced with a platinum one (covered with a platinum foil). Then, the fraction of the mouse hepatoma MH-22A cells, which died in the cell culture medium treated with a square-wave electric pulse, was determined. The pulse with a duration of 2 ms and an amplitude of 0.2 and 0.5 kV/cm (electric charge densities of 32 and 80 C/L, respectively) was used.

No detectable cytotoxicity toward MH-22A cells was observed when the stainless steel anode was replaced by the one made of platinum (the stainless steel cathode was still used) (Figure 4b). This allows us to rule out the cathodic primary reduction half-reactions and secondary chemical reactions caused by these primary ones as the main cause of the medium cytotoxicity when stainless steel electrodes are used. Therefore, the main source of the cell culture medium cytotoxicity comes from the primary oxidative half-reactions occurring at the interface between the stainless steel anode and electrolyte solution and/or subsequent processes caused by these reactions.

### 3.6. Release of Iron Ions from Stainless Steel Electrodes

To manufacture the stainless steel electrodes utilized throughout this study, the stainless steel of the austenitic family of the 300 series was used. The main constituents of this alloy were (average ±2SD, %): iron (69.87 ± 0.14), chromium (17.582 ± 0.075), nickel (10.28 ± 0.10), manganese (0.801 ± 0.055), titanium (0.80 ± 0.02), cobalt (0.294 ± 0.075), copper (0.19 ± 0.03), and molybdenum (0.070 ± 0.003).

The major constituent of stainless steel is iron, and, as a result, large amounts of ferric and/or ferrous ions can be released from the stainless steel anode (see Equations (10) and (11)) [15,17,18,19,20]. Thus, it is natural to attribute at least the part of the pulse-treated medium cytotoxicity to the iron ions released from the stainless steel electrodes. To confirm or rule out such a suggestion, first, the amount of iron ions released into the solution because of the exposure to a square-wave electric pulse had to be determined.

A 50 µL droplet of a 154 mM NaCl solution was placed between two stainless steel electrodes (inter-electrode distance was 2 mm) and subjected to a single square-wave electric pulse. The resistance was about 50 W. For each treatment, the total electric charge that had flown through the sample, *C*, and its density (charge per unit volume of the medium) were calculated using Equation (1). For example, the total electric charge that had flown through the sample during a 2 ms duration pulse with an amplitude of 240 V was about 9.6 mC, which gives us the charge density of 192 C/L.

In Figure 5, the obtained dependence of the concentration of iron ions released from the stainless steel anode into the solution on the charge density is shown (red circles). The vertical dashed red line in this graph indicates the level of the electric treatment at which 100% cytotoxicity of the electric pulse-treated cell culture medium for MH-22A cells is observed (see Figure 3a). It can be seen from Figure 5 that, the exposure of the sample with the charge density of 35 C/L led to the increase of the iron (Fe^2+^ & Fe^3+^) concentration in the sample by about 0.05 mM. To increase the iron concentration by 0.5 mM, the sample had to be treated with a charge density of about 190 C/L (Figure 5). This corresponds to a single square-wave pulse with a duration of 2 ms and an amplitude of 240 V (electric field strength of 1.2 kV/cm).

Similar results of the release of iron ions from the stainless-steel electrodes were reported by other groups as well [15,17]. Electric treatments with electric charge densities within the range of 120–1300 C/L caused the increase of the concentration of iron ions within the range of 0.05–3.7 mM [15,17]. Some examples of such results are shown in Figure 5. It should be mentioned that the amount of the electrode material that is transferred from electrodes into the solution depends strongly on the shape of the pulse [18].

### 3.7. Influence of Iron Ions on Cell Viability

To evaluate the role of iron ions in the cytotoxicity of a pulse-treated cell culture medium, the influence of the iron (Fe^2+^ and Fe^3+^) ions on the viability of mouse hepatoma MH–22A, rat glioma C6, and Chinese hamster ovary CHO cells was studied in more detail. The cells were seeded into the Petri dishes containing 2 mL of the culture medium supplemented with various concentrations of aqueous FeCl_2_ or FeCl_3_. Mouse hepatoma MH-22A cells were grown for 9–10 days, while rat glioma C6 and Chinese hamster ovary cells were grown for 6–7 days. Then, the cell viability was estimated by fixing the colonies with 96% ethanol, staining with a Gram’s crystal violet solution, and counting under a stereo microscope. The obtained dependences of the cell viability on the concentration of Fe^2+^ and Fe^3+^ ions added to the culture medium are presented in Figure 5.

Note that the abscissa axis shows not the actual concentration of Fe^3+^ ions in the medium, but the amount of Fe^3+^ ions that was added into the medium (to conform to the case of cell electroporation, when some amount of the iron ions is released from the electrodes). It should be mentioned that it is difficult to predict the actual concentration of free iron ions in the cell culture medium. First, this is because Fe^3+^ ions can easily precipitate [17,78]. Second, this is because some small amount of iron ions was already present in the medium, which was used to culture cells. In our experiments, cell culture medium was prepared from Dulbecco‘s modified Eagle‘s medium. It contains about 0.186 µM of ferric nitrate. Despite that the amount of ferric nitrate (Fe(NO_3_)_3_) in the medium is known; however, some fraction of iron ions (usually 25–30%) is bound to transferrin [79]. Although the exact concentration of transferrin in the medium is not known, in the medium supplemented with 10% of fetal-bovine serum, it can be as high as 570 µg/mL [79].

**Figure 6:**
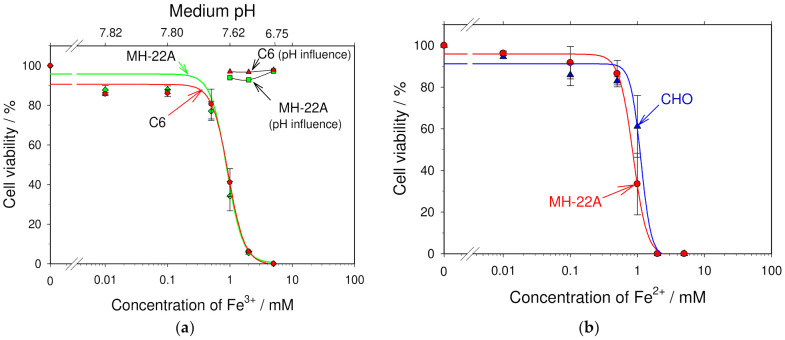
Influence of iron ions on cell viability. (**a**) The influence of Fe^3+^ ions on the viability of mouse hepatoma MH-22A and rat glioma C6 cells. The ordinate axis shows the cell viability and abscissa axis—what concentration of Fe^3+^ ions was added into the medium. The cells were grown in the medium supplemented with various amounts of aqueous FeCl_3_ and the cell viability was estimated by a colony–forming assay. The influence of the medium pH (upper abscissa) on the cell viability (ordinate) is also shown. (**b**) The influence of Fe^2+^ ions on the viability of mouse hepatoma MH-22A and Chinese hamster ovary CHO cells. The abscissa axis shows what concentration of Fe^2+^ ions was added into the medium. The cells were grown in the medium supplemented with various amounts of aqueous FeCl_2_, and the cell viability was estimated by a colony-forming assay.

It can be seen from Figure 6a that increasing the concentration of Fe^3+^ ions in the medium drastically reduced the viability of both types of cells studied here. More than 95% of mouse hepatoma MH-22A and rat glioma C6 cells died in the culture medium supplemented with 2 mM of Fe^3+^ ions (Figure 6a). LD_50_ of Fe^3+^ was 0.88 mM for both mouse hepatoma MH-22A and rat glioma C6 cells.

Similar dependences of the cell viability on the concentration of Fe^2+^ ions were obtained—Fe^2+^ ions were cytotoxic to mouse hepatoma MH-22A and Chinese hamster ovary cells when more than 0.5 mM of Fe^2+^ ions was added into the cell culture medium (Figure 6b). LD_50_ of Fe^2+^ was 0.84 and 1.09 mM for mouse hepatoma MH-22A and Chinese hamster ovary cells, respectively.

These results showing cytotoxicity of iron in concentrations within the millimolar range on mouse hepatoma MH-22A, rat glioma C6, and Chinese hamster ovary cells are in agreement with the data reported on other types of cells, e.g., DC-3F cells [15], human umbilical vein endothelial cells [80], and other ones [81].

However, it can be seen from Figure 6a that when the concentration of Fe^3+^ ions in the medium was 0.5 mM, the cell viability was reduced by only about 20%. Such a concentration of iron ions corresponds to the pre-treatment of the medium by a single square-wave electric pulse with a duration of 2 ms and an amplitude of about 1.2 kV/cm (electric charge density of 192 C/L) (see Figure 3). Meanwhile, none of the cells survived in the medium pre-treated with such an electric pulse (see Figure 2).

Recall that the values of the treatment intensity at which all cells are killed (when stainless steel electrodes are used) are in the range of 30–40 C/L. At such intensities, the concentration of iron ions increases by only about 0.04–0.06 mM (Figure 4). This clearly indicates that the iron ions released from the stainless steel anode are not the main direct cause of the cell death. Other plausible sources of the cytotoxicity have to be explored.

Often, during electroporation experiments, the cells are transferred to another medium or medium is diluted with a large volume of untreated medium immediately [82,83] or within 1–2 min [84] after an electric pulse. However, in some cases, especially in food processing experiments and when long series of pulses are used, the cells stay in the medium exposed to electric pulse/s (during the electric treatment and/or after it) for 10–20 min [53,85], several hours [86,87], or even days [39,88].

**Figure 7:**
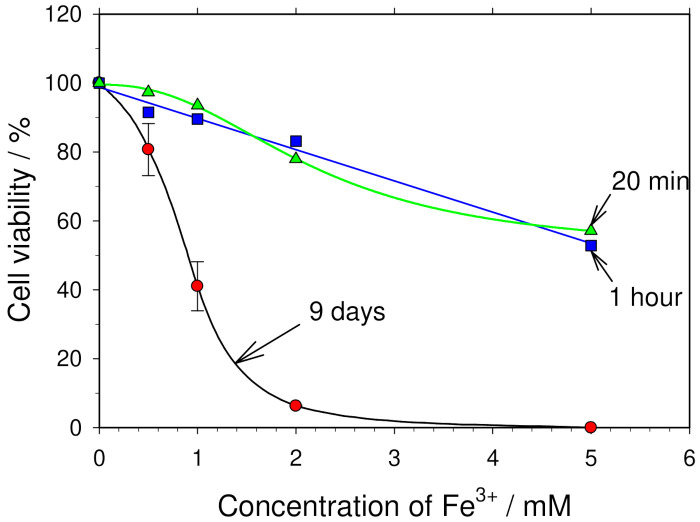
Influence of iron ions on the cell viability depending on the time of incubation. The influence of the Fe^3+^ ions on the viability of mouse hepatoma MH-22A cells (ordinate) for various durations of the incubation of cells in the medium supplemented with Fe^3+^. The abscissa axis shows what concentration of Fe^3+^ ions was added into the medium. The cell viability was estimated by a colony-forming assay.

It is natural to consider that the cytotoxicity of the iron ions should depend on the duration of the cell incubation in the medium supplemented with iron ions. To access the role of the duration of the exposure to iron ions on the cell viability, the dependence of the cell viability of the Fe^3+^ concentration was evaluated for the cases when cells were incubated in the growth medium supplemented with Fe^3+^ ions for 20 min and 1 h. The obtained dependences are shown in Figure 7. It can be seen from this figure that, only when the concentration of Fe^3+^ ions was higher than 1 mM (2–5 mM), even a short incubation for 20 min in the medium with Fe^3+^ ions reduced the cell viability substantially (by 20–45%).

Figure 7 shows that the influence of Fe^3+^ ions on cell viability can be neglected only if the post-pulse incubation of cells in the treated medium is short (20 min–1 h) and the amount of Fe^3+^ ions released from the anode is less than 0.5–1.0 mM. In other cases, in estimating the influence of the electric pulse on cell viability, it has to be taken into account that, besides an electric pulse exposure, the iron ions released from the electrodes can reduce cell viability as well.

### 3.8. Plausible Reasons of the Electric Pulse-Treated Cell Culture Medium

Cytotoxicity of iron is largely based on Fenton and Haber–Weiss reactions, where catalytic amounts of iron are sufficient to yield hydroxyl radicals (^•^HO) from superoxide (O_2_^•−^) and hydrogen peroxide (H_2_O_2_) [77]:

(16) $$Fe^{2+}+H_{2}O_{2}\to Fe^{3+}{+}^{•}HO+OH^{-},\;\;\;(\mathrm{Fenton}\;\mathrm{reaction})$$

(17) $$O_{2}^{•-}+H_{2}O_{2}\to O_{2}{+}^{•}HO+OH^{-},\;\;\;(\mathrm{Haber}\text{–}\mathrm{Weiss}\;\mathrm{reaction})$$

The hydroxyl radical is highly reactive with a half-life in an aqueous solution of less than 1 ns [89]. When produced in vivo, it reacts close to its site of formation. Production of ^•^HO close to DNA could lead to this radical reacting with DNA bases or the deoxyribose backbone of DNA to produce damaged bases or strand breaks [89].

In addition, the addition of FeCl_3_ into the culture medium slightly changed its pH. This is because iron ions behave as a Lewis acid and undergo spontaneous hydrolysis reactions [90]:

(18) $$Fe^{3+}+nH_{2}O\Leftrightarrow Fe{\left(OH\right)}_{n}^{3-n+}+nH^{+}$$

Due to this reaction, the pH is reduced at the anode. Production of H^+^ ions at the anode can partially be compensated by the production of HO^-^ ions at the cathode (reaction (2)). This can occur due to mixing of the solution in the experimental chamber, especially, in the case when the anode and cathode are close enough to each other, as diffusion of HO^−^ and H^+^ ions produced at the cathode and anode, respectively, cannot be neglected.

To ascertain whether the change of the medium pH can be a factor responsible for cell death in the pulse-treated medium, we determined how much the pH of the cell culture medium could change because of the iron ions added. In the upper axis of abscissa of Figure 6a, the pH of the medium, which corresponds to the amount of iron ions added (lower axis of abscissa), is shown. The medium pH decreases due to hydrolysis reactions in which Fe^3+^ ions participate (see Equation (18) [91]. In our experiments the medium pH was changed only slightly—it was reduced by 0.48 and 1.25 pH units, when 1 and 5 mM of FeCl_3_ was added into the cell culture medium, respectively [21].

In any case, we tested whether such a small decrease of the medium pH makes any influence on the viability of cells. The pH of a culture medium was reduced by adding the appropriate amounts of HCl (see Section 2 Materials and Methods). The percentage of viable mouse hepatoma MH-22A or rat glioma C6 cells was not affected when the pH was reduced down to 6.75, which corresponds to the supplementation of the medium with 5 mM of FeCl_3_ (Figure 6a, upper right corner).

Iron ions can also form complexes with various biomolecules, such as amino acids, nucleotides, physiological chelators, enzymes, and other proteins [17,92,93]. The specific complexes are very important because they determine whether the iron is available to participate in cell growth, to catalyze toxic reactions, or become non-available to the system. Free or ineffectively sequestered iron can be very toxic to cells. Properly complexed iron is available to support cell life and is essential to a cell culture system [93].

The results obtained in this study show that the effects caused by the iron ions released from the electrodes can be responsible only for a small fraction of the cytotoxicity of a cell culture medium treated with high-voltage pulses. Therefore, other sources of medium cytotoxicity should be taken into consideration. Most likely, the culture medium cytotoxicity is a sum of several factors affecting it in additive or synergistic ways.

Among plausible sources, hydrogen peroxide (H_2_O_2_) and other reactive oxygen species (ROS), which can be formed in the cell culture medium as a result of its treatment with a high-voltage pulse, have to be analyzed. For example, recently, it was shown that H_2_O_2_ can be formed in a cell-free media due to exposure to a succession of high-voltage pulses with a duration of 300 ns [94] and that H_2_O_2_ can be cytotoxic to cells [94]. However, concentrations of hydrogen peroxide observed (up to 1–2 µM) [94] were too low to be the only reason of cytotoxicity of the medium treated by electric pulses.

Hulsheger and Niemann have shown that when solutions containing chloride (Cl_2_) compounds were treated with electric pulses, hypochlorous acid (HClO) was produced as a result of a reaction of chlorine generated at the anode (see Equation (8)) with water [40]):

(19) $$Cl_{2}+H_{2}O\Leftrightarrow HClO+H^{+}+Cl^{-}$$

They suggested that this hypochloric acid contributed to the inactivation of bacteria *Escherichia coli* by pulsed electric fields [40].

In addition, iron ions are not the only ones that are released from stainless steel electrodes. The release of other constituents of stainless steel, such as, chromium, nickel, and manganese, during pulsed electric field treatment has been observed [18,19,76]. Meanwhile, some cytotoxicity of these materials has been reported [95,96,97].

The results obtained show that when utilizing high-voltage electric pulses for various applications, it should be taken into account that various electrochemical reactions/processes can take place in the system. Meanwhile, electrochemical processes occurring due to the action of extremely high-voltage pulses (up to 100 kV) are barely known. Therefore, electrochemists should help in investigating these processes, both theoretically and experimentally.

The authors hope that the results of the present work can be useful not only in understanding the mechanisms underlying cell death, which is caused by pulses of strong electric fields, but in other ways as well. It should help avoiding undesirable results of electrochemical processes occurring during high-voltage pulses, which are commonly used for cell electromanipulation procedures, as well as optimizing electroporation technology.

## 4. Conclusions

On the basis of the results obtained in the present study on the cytotoxicity of a cell culture medium pre-treated with a high-voltage pulse and iron (Fe^2+^ & Fe^3+^) ions, the following main conclusions can be drawn:

Products of electrolysis reactions can affect cell viability—a cell culture medium treated by an electric pulse is highly cytotoxic depending on the electrode material. The medium is much more cytotoxic when stainless steel electrodes are used in comparison to aluminum electrodes or a platinum anode.

The fraction of cells killed by a cell culture medium exposed to a high-voltage electric pulse is directly proportional to the total amount of the electric charge that has flown through the sample. The values of the treatment intensity at which all cells are killed (when stainless steel electrodes are used) are in the range of 30–40 C/L.

The main source of cell culture medium cytotoxicity comes from the primary oxidative anodic half-reactions (metal ions released) and/or subsequent processes caused by these reactions.

Although iron ions added to a culture medium in concentrations exceeding 0.5 mM are cytotoxic, iron ions released from a stainless steel anode are not the main direct cause of cell death induced by a medium treated with an electric pulse.

## Data Availability

Not applicable.
